# Rituximab Immunomonitoring Predicts Remission in Membranous Nephropathy

**DOI:** 10.3389/fimmu.2021.738788

**Published:** 2021-10-13

**Authors:** Maxime Teisseyre, Marion Cremoni, Sonia Boyer-Suavet, Thomas Crepin, Sylvia Benzaken, Kévin Zorzi, Vincent Esnault, Vesna Brglez, Barbara Seitz-Polski

**Affiliations:** ^1^ Laboratoire d’Immunologie, Centre Hospitalier Universitaire de Nice (CHU de Nice), Université Côte d’Azur, Nice, France; ^2^ Centre de Référence Maladies Rares Syndrome Néphrotique Idiopathique, Centre Hospitalier Universitaire de Nice (CHU de Nice), Université Côte d’Azur, Nice, France; ^3^ Service de Néphrologie-Dialyse-Transplantation, Centre Hospitalier Universitaire de Nice (CHU de Nice), Université Côte d’Azur, Nice, France; ^4^ Unité de Recherche Clinique de la Côte d’Azur (UR2CA), Université Côte d’Azur, Nice, France; ^5^ Centre Hospitalier Regional Universitaire de Besancon (CHU Besancon), Department of Nephrology, Dialysis, and Renal Transplantation, Besancon, France

**Keywords:** membranous nephropathy, nephrotic syndrome, autoimmunity, rituximab, chronic kidney disease rituximab immunomonitoring in membranous nephropathy

## Abstract

Primary membranous nephropathy (pMN) is an autoimmune kidney disease and a common cause of nephrotic syndrome in adults. Rituximab is becoming a first line therapy for patients with persistent nephrotic syndrome with proven safety and efficacy, achieving remission in 60%–80% of cases. For the remaining 20%–40% of patients there is an urgent need to identify early biomarkers of resistance to rituximab to adapt therapeutic management. In nephrotic patients, rituximab is found in the blood more transiently than in other autoimmune diseases without proteinuria, due to rituximab wasting in the urine. However, rituximab immunomonitoring is not routinely performed. We evaluated the predictive value of serum rituximab levels in patients with pMN three months after rituximab injection (month-3) on clinical remission rates six months (month-6) and 12 months (month-12) after injection and investigated predictive factors for serum rituximab levels at month-3. Sixty-eight patients treated with rituximab between July 2015 and January 2020 from two French nephrology centers were included. We identified residual rituximab levels at month-3 as a novel early predictor of remission at month-6 (*p* <0.0001) and month-12 (*p* = 0.001). Reduced likelihood of remission in patients with undetectable rituximab at month-3 was associated with lower serum albumin and higher anti-PLA2R1 titers at baseline and with lower serum albumin, higher proteinuria, higher CD19^+^ counts and higher anti-PLA2R1 titers during follow-up. In multivariate analysis, high baseline proteinuria and undetectable rituximab levels at month-3 were independent risk factors for treatment failure at month-6 and high baseline weight and undetectable rituximab levels at month-3 were independent risk factors for treatment failure at month-12. We identified serum albumin at baseline as a predictive factor for serum rituximab levels at month-3. Patients with serum albumin below 22.5 g/L at baseline had an 8.66-fold higher risk of having undetectable rituximab levels at month-3. Therefore, rituximab immunomonitoring in pMN patients treated with rituximab would allow the detection of patients at risk of treatment failure as early as month-3. Studies are needed to assess whether patients with low residual rituximab levels at month-3 may benefit from an early additional course of rituximab.

## Introduction

Primary membranous nephropathy (pMN) is an autoimmune disease affecting kidney glomerulus and the most common cause of nephrotic syndrome (NS) in non-diabetic adults. The course of the disease is highly variable, ranging from spontaneous remission to progressive chronic kidney disease. Histologically, pMN is characterized by subepithelial immune deposits containing immunoglobulins G (IgG) and complement fractions resulting in thickening of the glomerular basement membrane and the formation of spikes (1). The pathophysiology of pMN involves autoantibodies targeting podocyte proteins such as M-type phospholipase A2 receptor 1 (PLA2R1) and thrombospondin type-1 domain-containing 7A (THSD7A) in 70%–80% and 3%–5% of patients, respectively (2, 3). Immune complex deposits are responsible for the activation of the complement cascade and podocyte damage (4–6). The pathogenicity of anti-PLA2R1 and anti-THSD7A autoantibodies has been demonstrated *in vitro* and *in vivo* (4, 7, 8). The recognition of pMN as an autoantibody-mediated disease has promoted the use of immunosuppressive drugs. Rituximab – a chimeric monoclonal antibody targeting CD20 – can trigger B cell death by apoptosis, complement-mediated cytotoxicity and antibody-dependent cellular cytotoxicity leading to an elimination of autoantibodies (9–11). Rituximab was first developed for the treatment of hematological malignancies, but is now used to treat many immune-mediated diseases (12). Rituximab is progressively becoming a first line therapy for pMN patients with proven safety and efficacy, achieving remission in 60%–80% of patients (13–15). However, for the remaining 20%–40% of patients there is an urgent need to identify early biomarkers of resistance to rituximab in order to adapt therapeutic management. Some patients with pMN may develop anti-rituximab antibodies that may decrease the effectiveness of the treatment (16). In these cases obinutuzumab and ofatumumab have been shown to be effective (17–20). Other patients are undertreated because of the highly variable bioavailability of rituximab in nephrotic patients (21). In nephrotic patients, rituximab – which binds to albumin – can be eliminated in the urine, thus rituximab is found in the blood more transiently than in other autoimmune diseases treated with rituximab without proteinuria (21, 22). There is still uncertainty about which rituximab protocol to use in nephrotic patients. Patients with the shortest exposure to rituximab could benefit from additional courses of rituximab to increase their likelihood of clinical remission. However, rituximab immunomonitoring is not yet routinely performed in patients with pMN.

The aims of this study were: (i) to evaluate the predictive value of serum rituximab levels in patients with pMN three months after rituximab injection (month-3) on clinical remission rates six months (month-6) and 12 months (month-12) after rituximab injection and (ii) to establish predictive factors for undetectable serum rituximab levels at month-3.

## Material and Methods

### Study Participants

Sixty-eight patients with pMN from a prospective cohort were included (NCT02199145). Patients were enrolled from two French nephrology centers from July 2015 to January 2020. The inclusion criteria were (1) biopsy-proven diagnosis of membranous nephropathy; (2) primary membranous nephropathy defined by the absence of concomitant autoimmune disease, negative hepatitis B and C serologies, and negative cancer workup; (3) rituximab treatment with two 1 g infusions two weeks apart; and (4) serum samples available at month-3. Patients should not receive any other immunosuppressive therapy at the same time as rituximab. The study protocol conformed to the ethical guidelines of the 1975 Declaration of Helsinki and was approved by the appropriate institutional review committee. Written informed consent was obtained from participants prior to inclusion in the study.

### Outcome

Clinical remission was evaluated at month-6 and month-12. Clinical remission was defined according to the 2012 Kidney Disease: Improving Global Outcomes (KDIGO) guidelines (23). Complete remission was defined by a urinary protein-to-creatinine ratio < 0.3 g/d, accompanied by a normal serum albumin concentration and a preserved kidney function. Partial remission was defined by urinary protein-to-creatinine ratio < 3.5 g/d with 50% reduction of proteinuria, accompanied by an improvement or normalization of the serum albumin concentration and preserved kidney function. Treatment failure was defined by the lack of clinical remission.

### Detection of Anti-PLA2R1 and Anti-THSD7A Antibodies

Serum levels of total IgG anti-PLA2R1 antibodies were measured by the ELISA test developed by EUROIMMUN (Medizinische Labordiagnostika AG, Lübeck, Germany). Participants were considered as anti-PLA2R1–positive when levels were >14 RU/mL.

Total IgG anti-THSD7A antibodies were measured by the indirect immunofluorescence test developed by EUROIMMUN (Medizinische Labordiagnostika AG, Lübeck, Germany).

### Rituximab Immunomonitoring by ELISA

Serum levels of rituximab were measured by ELISA according to the manufacturer’s instructions (LISA-TRACKER Duo Rituximab; Theradiag, Croissy Beaubourg, France). This assay measures only free rituximab. The limit of detection defined by the manufacturer was 2 µg/mL. Rituximab levels were measured 3 months after the last infusion. This time interval was chosen based on a previous bioavailability study (21).

### Anti-Rituximab Antibodies Detection

Anti-rituximab antibodies were detected by ELISA (LISA-TRACKER, Theradiag, Croissy Beaubourg, France). The limit of detection for anti-rituximab antibodies defined by the manufacturer was 5 ng/mL.

### Statistical Analyses

Categorical data were described as frequency and percentage and were compared using Fisher’s exact test. Quantitative data were described as median and interquartile range and were compared using Wilcoxon-Mann-Whitney test. Survival curves were assessed by the Kaplan-Meier method and compared with the log-rank test. A *P-*value < 0.1 was used to select variables that were entered into a single multivariate logistic model with backward selection (threshold = 0.05). We performed a receiver operating characteristic curve (ROC) analysis to assess the optimal threshold of predictive factors identified in multivariate analysis that could predict the risk of undetectable rituximab levels at month-3. A *P-*value < 0.05 indicated statistical significance. Statistical analyses were performed using GraphPad Prism 8.4.3 (GraphPad Software Inc., San Diego, CA) and SAS Enterprise Guide 7.1 (SAS Institute Inc., Cary, NC).

## Results

### Population Characteristics

Sixty-eight patients with pMN treated with rituximab (two 1 g infusions two weeks apart) were included. A supportive therapy with angiotensin-converting enzyme inhibitor or angiotensin receptor blocker was consistently associated with rituximab. **Table 1** shows the characteristics of the study cohort at baseline (i.e. the day of rituximab injection) and during follow-up.

**Table 1:** Characterization of patients at baseline (i.e. the day of rituximab injection) and during follow-up.

Variables	All patients (n = 68)	Patients with serum rituximab level < 2µg/mL at month-3 (n = 38)	Patients with serum rituximab level > 2µg/mL at month-3 (n = 30)	*P-*value
**Characteristics at baseline**				
Age (years)	58.5 [49.0–67.2]	57.5 [44.0–67.5]	60.5 [50.5–68.0]	0.3
Gender (Female/Male)	19/49	14/24	5/25	0.1
Weight (kg)	76.6 [67.4-84.0]	76.3 [66.6-83.6]	77.0 [67.9-86.0]	1
UP (g/d)	6.0 [4.3–8.9]	7.0 [4.9–10.1]	5.5 [3.9–7.1]	0.07
Serum creatinine (µmol/L)	120 [87–151]	119 [83–138]	137 [90–183]	0.1
Serum albumin (g/L)	22.6 [16.0–29.0]	20.2 [14.1–24.6]	26.6 [22.0–31.7]	0.001
CD19^+^ count (cell/µL)	186.5 [123.5–270.0]	208.0 [138.0–280.0]	142.5 [77.2–215.5]	0.2
Etiology				0.2
Anti-PLA2R1-associated pMN	62 (91%)	35 (92%)	27 (90%)	
Anti-THSD7A-associated pMN	2 (3%)	0 (0%)	2 (7%)	
Double negative patients	4 (6%)	3 (8%)	1 (3%)	
Anti-PLA2R1 Ab titer (RU/mL)	149 [57–303]	184 [71–489]	89 [20–173]	0.01
Anti-proteinuric treatment	68 (100%)	38 (100%)	30 (100%)	1
**Characteristics at month-3**				
UP (g/d)	3.0 [1.5–5.9]	5.4 [2.4–8.1]	1.8 [1.1–3.2]	<0.0001
Serum creatinine (µmol/L)	110 [92–142]	110 [87–139]	105 [92–166]	0.7
Serum albumin (g/L)	29.0 [23.0–35.0]	24.6 [17.7–31.3]	35.0 [29.5–37.3]	0.0002
CD19^+^ count (cell/µL)	2.0 [0.0–7.0]	2.5 [0.9–18.5]	0.0 [0.0–2.0]	0.005
Anti-PLA2R1 Ab titer (RU/mL)	5 [0–23]	19 [5–63]	0 [0–4]	<0.0001
Patients with anti-RTX Ab	0 (0%)	0 (0%)	0 (0%)	1
**Characteristics at month-6**				
UP (g/d)	1.7 [1.0–4.7]	4.5 [1.7–8.0]	1.2 [0.6–1.7]	<0.0001
Serum creatinine (µmol/L)	107 [89–137]	110 [90–139]	104 [85–142]	0.7
Serum albumin (g/L)	32.5 [25.5–37.0]	29.0 [22.2–33.6]	37.0 [32.8–39.2]	<0.0001
CD19^+^ count (cell/µL)	22.5 [3.0–52.2]	41.0 [19.0–127.0]	2.0 [1.0–33.0]	0.0009
Anti-PLA2R1 Ab titer (RU/mL)	3 [0–24]	15 [2–80]	0 [0–4]	<0.0001
Patients with anti-RTX Ab	6 (9%)	4 (11%)	2 (7%)	0.7
**Clinical outcome**				
Remission at month-6	36 (53%)	12 (32%)	24 (80%)	<0.0001
Remission at month-12	41 (60%)	16 (42%)	25 (83%)	0.001

Anti-PLA2R1 Ab, anti-Phospholipase A2 Receptor 1 antibody; Anti-THSD7A Ab, anti-Thrombospondin Type 1 Domain Containing 7A antibody; Anti-RTX Ab, anti-rituximab antibody; pMN, primary membranous nephropathy; UP, 24-hour urinary protein excretion.

Serum rituximab levels were measured at month-3. In 38 patients (56%), rituximab was undetectable in the serum (i.e. < 2 µg/mL) at month-3. In 30 patients (44%), serum rituximab was over 2 µg/mL at month-3. At baseline, in patients with undetectable serum rituximab levels at month-3, anti-PLA2R1 titers were higher and serum albumin levels were lower than in patients with serum rituximab levels > 2 µg/mL at month-3 (**Table 1**). Proteinuria tended to be higher in patients with undetectable serum rituximab levels at month-3, although this did not reach statistical significance (**Table 1**). All other baseline characteristics were similar, including age, gender, weight, type of autoantibodies, serum creatinine levels and CD19^+^ cell counts.

During follow-up in patients with undetectable serum rituximab levels at month-3, anti-PLA2R1 titers were higher at month-3 and month-6; proteinuria was higher at month-3 and month-6; and serum albumin levels were lower at month-3 and month-6 than in patients with serum rituximab levels > 2 µg/mL at month-3 (**Table 1**). B cells re-emerged more quickly in patients with undetectable serum rituximab levels at month-3 than in patients with serum rituximab levels > 2 µg/mL at month-3 (**Table 1**).

Anti-rituximab antibodies were never detected at month-3, and were detected at month-6 in 4 of 38 patients (11%) with undetectable serum rituximab levels at month-3 and 2 of 30 patients (7%) with serum rituximab levels > 2 µg/mL at month-3 (**Table 1**).

### Outcome

**Figure 1:**
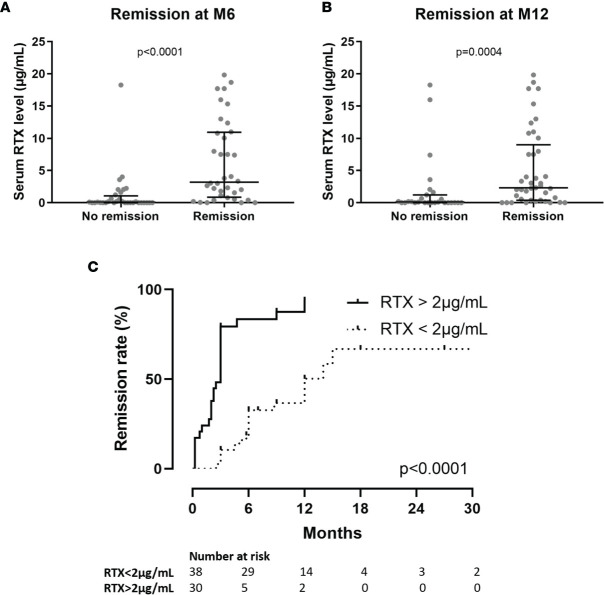
**(A)** Serum rituximab (RTX) levels at month-3 (M3) according to clinical status at month-6 (M6). **(B)** Serum rituximab (RTX) levels at month-3 (M3) according to clinical status at month-12 (M12). **(C)** Time from initiation of rituximab treatment to remission of nephrotic syndrome according to serum rituximab levels at month-3. M3, month-3 after rituximab injection; M6, month-6 after rituximab injection; M12, month-12 after rituximab injection; RTX, rituximab; UP, 24-hour urinary protein excretion.

Clinical remission was analyzed at month-6 and month-12. Clinical remission (partial or complete) was obtained in 36 of 68 patients (53%) at month-6 and in 41 of 68 patients (60%) at month-12 (**Table 1**). Serum rituximab levels at month-3 were significantly lower in patients who failed to achieve clinical remission at month-6 (0.06 µg/mL [IQR, 0.1–1.1] *versus* 3.2 µg/mL [IQR, 0.9–11.0]; *p* < 0.0001) and at month-12 (0.1 µg/mL [IQR, 0.0–1.2] *versus* 2.3 µg/mL [IQR 0.4–9.0]; *p* = 0.0004) (**Figures 1A, B**). Patients with serum rituximab levels > 2 µg/mL at month-3 achieved clinical remission more frequently at month-6 and month-12 than patients with undetectable serum rituximab levels at month-3 (**Table 1**). Kaplan–Meier analysis demonstrates faster time to clinical remission when serum rituximab levels are over 2 µg/mL at month-3 (**Figure 1C**). After pairing data for age, gender, weight and anti-PLA2R1 titers at month-3, with propensity score matching using logit model, serum rituximab levels at month-3 predicted remission at month-6 (*p* = 0.02 using McNemar’s test). In multivariate analysis, an undetectable serum rituximab level at month-3 and high proteinuria at baseline were independent risk factors for treatment failure at month-6 (hazard ratio (HR), 12.00; 95% confidence interval (CI), 2.77–52.99; *p* = 0.007 and HR, 1.37; 95% CI, 1.08–1.73; *p* = 0.02, respectively) (**Table 2**); and an undetectable serum rituximab level at month-3 and high weight at baseline were independent risk factors for treatment failure at month-12 (HR, 10.98; 95% CI, 2.58–46.72; *p* = 0.01 and HR, 1.05; 95 % CI, 1.01–1.09; *p* = 0.02, respectively) (**Table 3**).

**Table 2:** Univariate and multivariate analysis of variables associated with clinical remission at month-6.

Variables	Remission at month-6 (n=36)	No remission at month-6 (n=32)	Univariate *P-*value	Multivariate *P-*value
Age (years)	58.0 [46.0–67.7]	58.5 [49.2–67.7]	0.7	
Gender (Female/Male)	9/27	10/22	0.6	
Weight (kg) at baseline*	74.3 [66.0–82.0]	77.3 [70.7-89.6]	0.1	0.1
UP (g/d) at baseline*	5.0 [4.0–7.0]	7.8 [5.8–12.2]	0.0001	0.02
Serum creatinine (µmol/L) at baseline	119 [86–151]	121 [87–163]	0.7	
Serum albumin (g/L) at baseline*	25.5 [20.7–30.2]	16.6 [12.5–23.0]	<0.0001	0.8
CD19^+^ count (cell/µL) at baseline	158.5 [112.8–277.5]	208.0 [122.0–291.0]	0.3	
CD19^+^ count (cell/µL) at month-3*	0.0 [0.0–2.7]	3.0 [1.0–25.0]	0.002	0.3
Etiology			0.2	
Anti-PLA2R1-associated pMN	33 (92%)	29 (91%)		
Anti-THSD7A-associated pMN	0 (0%)	2 (6%)		
Double negative patients	3 (8%)	1 (3%)		
Anti-PLA2R1 Ab titer (RU/mL) at baseline*	89 [21–173]	218 [76–561]	0.002	0.9
Patient with RTX <2µg/mL at month-3*	12 (33%)	26 (81%)	<0.0001	0.007

*Variables with p < 0.1 in univariate analysis tested into the multivariate logistic regression model.

Anti-PLA2R1 Ab, anti-Phospholipase A2 Receptor 1 antibody; Anti-THSD7A Ab, anti-Thrombospondin Type 1 Domain Containing 7A antibody; pMN, primary membranous nephropathy; RTX, rituximab; UP, 24-hour urinary protein excretion.

**Table 3:** Univariate and multivariate analysis of variables associated with clinical remission at month-12.

Variables	Remission at month-12 (n=41)	No remission at month-12 (n=27)	Univariate *P-*value	Multivariate *P-*value
Age (years)	60.0 [50.0–68.0]	56.0 [42.0–67.0]	0.3	
Gender (Female/Male)	11/30	8/19	1	
Weight (kg) at baseline*	71.5 [66.0–82.0]	78.0 [74.0–89.2]	0.06	0.02
UP (g/d) at baseline*	5.2 [4.0–7.1]	7.4 [5.6–11.9]	0.005	0.2
Serum creatinine (µmol/L) at baseline	118 [87–149]	123 [87–184]	0.4	
Serum albumin (g/L) at baseline*	24.8 [20.2–29.4]	16.0 [13.0–22.1]	0.001	0.7
CD19^+^ count (cell/µL) at baseline	186.0 [125.0–285.0]	185.0 [88.7–263.8]	0.9	
CD19^+^ count (cell/µL) at month-3*	0.0 [0.0–2.7]	3.0 [1.0–16.0]	0.005	0.9
Etiology			0.8	
Anti-PLA2R1-associated pMN	37 (90%)	25 (93%)		
Anti-THSD7A-associated pMN	1 (2%)	1 (3%)		
Double negative patients	3 (8%)	1 (3%)		
Anti-PLA2R1 Ab titer (RU/mL) at baseline*	86 [21–188]	226 [110–561]	0.001	0.5
Patient with RTX <2µg/mL at month-3*	16 (26%)	22 (85%)	0.001	0.01

*Variables with p < 0.1 in univariate analysis tested into the multivariate logistic regression model.

Anti-PLA2R1 Ab, anti-Phospholipase A2 Receptor 1 antibody; Anti-THSD7A Ab, anti-Thrombospondin Type 1 Domain Containing 7A antibody; pMN, primary membranous nephropathy; RTX, rituximab; UP, 24-hour urinary protein excretion.

**Figure 2:**
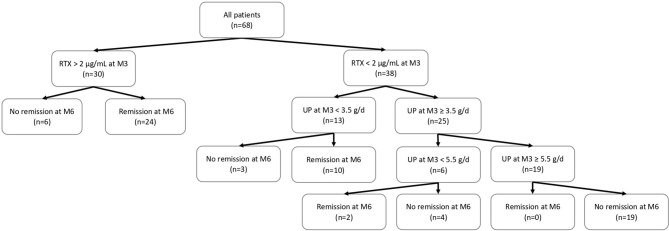
Flow chart of patients included in this study and outcome according to serum rituximab levels at month-3. M3, month-3 after rituximab injection; M6, month-6 after rituximab injection; RTX, rituximab; UP, 24-hour urinary protein excretion.

For patients with undetectable serum rituximab levels at month-3: (i) clinical remission was never achieved at month-6 if proteinuria was greater than 5.5 g/d at month-3; (ii) clinical remission was achieved at month-6 in 33% of cases if proteinuria was between 3.5 g/d and 5.5 g/d at month-3; and (iii) clinical remission was achieved at month-6 in 77% of cases if proteinuria was less than 3.5 g/d at month-3 (**Figure 2**).

Regarding cases who developed anti-rituximab antibodies, 4 of 6 patients (67%) did not achieve clinical remission at either month-6 or month-12. Two patients with anti-rituximab antibodies (1 with undetectable serum rituximab at month-3 and 1 with serum rituximab > 2 µg/mL at month-3) achieved partial clinical remission at month-6.

We then investigated the impact of rituximab levels on clinical outcome in patients with severe nephrotic syndrome, i.e. with baseline proteinuria exceeding the median value (6 g/d). Among these patients, 20 patients had undetectable rituximab levels at month-3 and 14 patients had rituximab levels > 2 µg/ml at month-3. Patients with serum rituximab levels > 2 µg/ml at month-3 achieved clinical remission more frequently at month-6 and month-12 than patients with undetectable serum rituximab levels at month-3 (at month-6: 64% *versus* 10%, *p* = 0.002 and at month-12: 71% *versus* 30%, *p* = 0.03).

### Factors Associated With Undetectable Serum Rituximab Levels at Month-3

In multivariate analysis, only baseline serum albumin level was associated with the risk of having an undetectable serum rituximab level at month-3 (HR, 0.89; 95% CI, 0.82 –0.97; *p* =0.005) (**Table 4**). Using ROC curves, we determined a threshold of albuminemia at baseline of 22.5 g/L that was associated with 8.66-fold (95% CI, 2.90–25.87) higher risk of undetectable serum rituximab levels at month-3 with a sensitivity of 77% (95% CI, 59–88%) and a specificity of 71% (95% CI, 55–83%) (air under curve (AUC) = 0.72; *p* = 0.002).

**Table 4:** Univariate and multivariate analysis of variables associated with serum rituximab levels at month-3.

Variables	Patients with serum rituximab level < 2µg/mL at month-3 (n = 38)	Patients with serum rituximab level > 2µg/mL at month-3 (n = 30)	Univariate *P-*value	Multivariate *P-*value
**Characteristics at baseline**				
Age (years)	57.5 [44.0–67.5]	60.5 [50.5–68.0]	0.3	
Gender (Female/Male)*	14/24	5/25	0.1	0.3
Weight (kg)	76.3 [66.6-83.6]	77.0 [67.9-86.0]	1	
UP (g/d)*	7.0 [4.9–10.1]	5.5 [3.9–7.1]	0.07	0.2
Serum creatinine (µmol/L)*	119 [83–138]	137 [90–183]	0.1	0.3
Serum albumin (g/L)*	20.2 [14.1–24.6]	26.6 [22.0–31.7]	0.001	0.005
CD19^+^ count (cell/µL)	208.0 [138.0–280.0]	142.5 [77.2–215.5]	0.2	
Etiology			0.2	
Anti-PLA2R1-associated pMN	35 (92.%)	27 (90%)		
Anti-THSD7A-associated pMN	0 (0%)	2 (7%)		
Double negative patients	3 (8%)	1 (3%)		
Anti-PLA2R1 Ab titer (RU/mL)*	184 [71–489]	89 [20–173]	0.01	0.5

*Variables with p < 0.1 in univariate analysis tested into the multivariate logistic regression model.

Anti-PLA2R1 Ab, anti-Phospholipase A2 Receptor 1 antibody; Anti-THSD7A Ab, anti-Thrombospondin Type 1 Domain Containing 7A antibody; pMN, primary membranous nephropathy; UP, 24-hour urinary protein excretion.

## Discussion

The first aim of this study was to evaluate the predictive value of serum rituximab levels at month-3 in patients with pMN on clinical remission rates at month-6 and month-12 after rituximab injection. We observed that rituximab underdosing is not uncommon, with 56% of patients having undetectable serum rituximab levels at month-3. Undetectable serum rituximab levels at month-3 were associated with lower clinical remission rates at month-6 and month-12 and with a longer time to achieve clinical remission. Undetectable serum rituximab levels at month-3 were also associated with higher PLA2R1 titers and lower serum albumin levels at baseline and with higher PLA2R1 titers, higher proteinuria, higher CD19^+^ counts and lower serum albumin levels at month-3 and month-6. These results suggest that patients with severe NS have a shorter exposure to rituximab – likely due to rituximab wasting in the urine – compared to patients with a mild NS or without NS, leading to a decrease in the effectiveness of B cell depletion and therefore to a higher rate of treatment failure. This is consistent with our previous results showing that higher residual serum rituximab concentrations at month-3 were significantly correlated with higher B cell depletion and lower requirement for repeat doses of rituximab (21).

The second aim of this study was to identify factors associated with undetectable serum rituximab levels at month-3. Little attention has been paid to rituximab pharmacokinetics in nephrotic patients. Antibodies – including rituximab – interact with the neonatal Fc receptor for IgG (FcRn) present on epithelial cells, endothelial cells, monocytes and/or macrophages, and dendritic cells (24). FcRn protects antibodies from degradation by lysosomes and thus reduces antibody clearance by allowing recycling into the cellular environment (25, 26). However, despite rituximab recycling, nephrotic patients have a shorter exposure to rituximab compared to a population without proteinuria, due to the clearance of rituximab in the urine (22). Fervenza *et al.* have demonstrated a shorter rituximab half-life in patients with pMN compared to patients with rheumatoid arthritis (11.5 days *versus* 18.0 days) treated with four doses of 375 mg/m^2^ (day 1, day 8, day 15, and day 22) (15). More recently, we have shown that residual rituximab levels at month-3 were significantly lower in patients with pMN compared to myasthenia gravis patients without proteinuria matched for age, gender and weight and treated with a similar treatment regimen (21). In addition to urinary excretion of rituximab, internalization and destruction of rituximab by target B cells (27, 28) and anti-rituximab antibodies (16, 20) may also contribute to decreased residual rituximab levels. Here, we identified baseline serum albumin level as a predictive factor for residual serum rituximab levels at month 3. Patients with severe NS and especially with serum albumin below 22.5 g/L at baseline were more likely to have an undetectable serum rituximab level at month-3. Previously, we have shown that a high-dose rituximab protocol (two perfusions of 1 g two weeks apart) was more effective than a low-dose rituximab protocol (two perfusions of 375 mg/m^2^ one week apart) in achieving clinical remission at month-6 in patients with pMN (29). The median residual rituximab level at month-3 was also higher with the high-dose rituximab protocol (29). Our present results suggest that this regimen of rituximab may still be insufficient in patients with severe NS. It has recently been shown that reinfusion of rituximab can induce remission in patients considered refractory to rituximab (20, 30). Therefore, the management of pMN patients treated with rituximab should be individually adjusted by rituximab immunomonitoring. For patients with undetectable serum rituximab levels at month-3 and active NS, additional rituximab injections should be planned and supportive therapy optimized to obtain higher serum rituximab levels and thereby increase the likelihood of remission and reduce time with active NS. Additional doses of rituximab should be considered as early as month-3 in patients with proteinuria greater than 5.5 g/d at month-3 as we have shown that these patients never achieve remission at month-6. However, it seems acceptable to wait until month-6 for patients with proteinuria between 3.5 g/d and 5.5 g/d at month-3 as we have shown that 33% of these patients achieve remission at month-6. The addition of a calcineurin inhibitor (CNI) to rituximab could also be discussed (31). In addition to their immunosuppressive effect, CNIs may decrease the urinary loss of rituximab by causing afferent and efferent glomerular arteriolar vasoconstriction. However, this combination is likely to result in enhanced immunosuppression with concomitant adverse events.

Recently, a study has shown that rituximab was less effective than cyclophosphamide in inducing immunological remission in patients with the highest anti-PLA2R1 titers (32). However, patients with the highest anti-PLA2R1 titers were more likely to have severe NS and thus more likely to be underdosed in rituximab (33–35). In these patients, a personalized management based on the rituximab immunomonitoring at month-3 may increase the effectiveness of rituximab and thus be as effective as cyclophosphamide but with less toxicity.

This study has several limitations. First, it is a retrospective analysis of a prospective cohort but we used systematically collected prospective data and samples. Second, the number of participants is relatively small. Third, patients with undetectable serum rituximab levels at month-3 had more severe disease at baseline, which may suggest that initial disease severity may contribute to their worse outcome. However, in multivariate analysis, an undetectable serum rituximab level at month-3 was an independent risk factor for treatment failure at months 6 and 12. Furthermore, by comparing the outcome of patients with detectable or undetectable rituximab levels in the group of patients with baseline proteinuria > 6.0 g/d – these patients are expected to have a similarly severe nephrotic syndrome – we demonstrated that rituximab levels were still predictive of clinical remission at months 6 and 12. Thus, these results show that rituximab levels at month-3 are predictive of clinical remission, regardless of the initial severity of the disease.

To conclude, in pMN patients treated with rituximab, rituximab immumonitoring at month-3 might be a useful biomarker to adapt therapeutic management in addition to CD19^+^ cell count monitoring, anti-rituximab antibodies monitoring (16, 20), anti-PLA2R1 antibody titer (36) and PLA2R1 epitope spreading (37). Personalized management may increase the efficacy of rituximab in patients with severe NS and may dispense the use of cyclophosphamide. Prospective studies are needed to compare the personalized management of patients with pMN based on rituximab immunomonitoring to conventional management.

## Data Availability Statement

The raw data supporting the conclusions of this article will be made available by the authors, without undue reservation.

## Ethics Statement

The studies involving human participants were reviewed and approved by NCT02199145. The patients/participants provided their written informed consent to participate in this study.

## Author Contributions

BS-P and VB provided the research idea and study design. MT, VB, BS-P, and KZ analyzed and interpreted the data. BS-P, VE, TC, MC, and SB-S provided medical oversight. VB and SB performed rituximab measurement and anti-rituximab antibody detection. MT, BS-P, and VB drafted and revised the manuscript. BS-P and VB contributed equally to this work. All authors contributed to the article and approved the submitted version.

## Funding

This study was academic, funded by Nice University Hospital.

## Conflict of Interest

The authors declare that the research was conducted in the absence of any commercial or financial relationships that could be construed as a potential conflict of interest.

## Publisher’s Note

All claims expressed in this article are solely those of the authors and do not necessarily represent those of their affiliated organizations, or those of the publisher, the editors and the reviewers. Any product that may be evaluated in this article, or claim that may be made by its manufacturer, is not guaranteed or endorsed by the publisher.

## References

[1] Couser WG. Primary Membranous Nephropathy. Clin J Am Soc Nephrol CJASN (2017) 12(6):983–97. doi: 10.2215/CJN.11761116 PMC5460716 28550082

[2] Beck LH Bonegio RGB Lambeau G Beck DM Powell DW Cummins TD. M-Type Phospholipase A2 Receptor as Target Antigen in Idiopathic Membranous Nephropathy. N Engl J Med (2009) 361(1):11–21. doi: 10.1056/NEJMoa0810457 19571279 PMC2762083

[3] Tomas NM Beck LH Meyer-Schwesinger C Seitz-Polski B Ma H Zahner G. Thrombospondin Type-1 Domain-Containing 7A in Idiopathic Membranous Nephropathy. N Engl J Med (2014) 371(24):2277–87. doi: 10.1056/NEJMoa1409354 PMC4278759 25394321

[4] Lateb M Ouahmi H Payré C Brglez V Zorzi K Dolla G. Anti-PLA2R1 Antibodies Containing Sera Induce In Vitro Cytotoxicity Mediated by Complement Activation. J Immunol Res (2019) 2019:1324804. doi: 10.1155/2019/1324804 32083137 PMC7012209

[5] Brglez V Boyer-Suavet S Seitz-Polski B. Complement Pathways in Membranous Nephropathy: Complex and Multifactorial. Kidney Int Rep (2020) 5(5):572–4. doi: 10.1016/j.ekir.2020.02.1033 PMC7210742 32406418

[6] Ronco P Debiec H. Molecular Pathogenesis of Membranous Nephropathy. Annu Rev Pathol (2019) 15:287–313. doi: 10.1146/annurev-pathol-020117-043811 31622560

[7] Tomas NM Hoxha E Reinicke AT Fester L Helmchen U Gerth J. Autoantibodies Against Thrombospondin Type 1 Domain-Containing 7A Induce Membranous Nephropathy. J Clin Invest (2016) 126(7):2519–32. doi: 10.1172/JCI85265 PMC4922694 27214550

[8] Meyer-Schwesinger C Tomas NM Dehde S Seifert L Hermans-Borgmeyer I Wiech T. A Novel Mouse Model of Phospholipase A2 Receptor 1-Associated Membranous Nephropathy Mimics Podocyte Injury in Patients. Kidney Int (2020) 97(5):913–9. doi: 10.1016/j.kint.2019.10.022 32033781

[9] Manches O Lui G Chaperot L Gressin R Molens J-P Jacob M-C. In Vitro Mechanisms of Action of Rituximab on Primary Non-Hodgkin Lymphomas. Blood (2003) 101(3):949–54. doi: 10.1182/blood-2002-02-0469 12393572

[10] Bonavida B. Rituximab-Induced Inhibition of Antiapoptotic Cell Survival Pathways: Implications in Chemo/Immunoresistance, Rituximab Unresponsiveness, Prognostic and Novel Therapeutic Interventions. Oncogene (2007) 26(25):3629–36. doi: 10.1038/sj.onc.1210365 17530016

[11] Wang S-Y Racila E Taylor RP Weiner GJ. NK-Cell Activation and Antibody-Dependent Cellular Cytotoxicity Induced by Rituximab-Coated Target Cells Is Inhibited by the C3b Component of Complement. Blood (2008) 111(3):1456–63. doi: 10.1182/blood-2007-02-074716 PMC2214766 18024795

[12] Lim SH Beers SA French RR Johnson PWM Glennie MJ Cragg MS. Anti-CD20 Monoclonal Antibodies: Historical and Future Perspectives. Haematologica (2010) 95(1):135–43. doi: 10.3324/haematol.2008.001628 PMC2805725 19773256

[13] Remuzzi G Chiurchiu C Abbate M Brusegan V Bontempelli M Ruggenenti P. Rituximab for Idiopathic Membranous Nephropathy. Lancet Lond Engl (2002) 360(9337):923–4. doi: 10.1016/S0140-6736(02)11042-7 12354476

[14] Fervenza FC Cosio FG Erickson SB Specks U Herzenberg AM Dillon JJ. Rituximab Treatment of Idiopathic Membranous Nephropathy. Kidney Int (2008) 73(1):117–25. doi: 10.1038/sj.ki.5002628 17943078

[15] Fervenza FC Abraham RS Erickson SB Irazabal MV Eirin A Specks U. Rituximab Therapy in Idiopathic Membranous Nephropathy: A 2-Year Study. Clin J Am Soc Nephrol CJASN (2010) 5(12):2188–98. doi: 10.2215/CJN.05080610 PMC2994079 20705965

[16] Boyer-Suavet S Andreani M Lateb M Savenkoff B Brglez V Benzaken S. Neutralizing Anti-Rituximab Antibodies and Relapse in Membranous Nephropathy Treated With Rituximab. Front Immunol (2019) 10:3069. doi: 10.3389/fimmu.2019.03069 31998325 PMC6970431

[17] Sethi S Kumar S Lim K Jordan SC. Obinutuzumab is Effective for the Treatment of Refractory Membranous Nephropathy. Kidney Int Rep (2020) 5(9):1515–8. doi: 10.1016/j.ekir.2020.06.030 PMC7486170 32954076

[18] Klomjit N Fervenza FC Zand L. Successful Treatment of Patients With Refractory PLA2R-Associated Membranous Nephropathy With Obinutuzumab: A Report of 3 Cases. Am J Kidney Dis (2020) 76(6):883–8. doi: 10.1016/j.ekir.2020.02.433 32311405

[19] Podestà MA Gennarini A Portalupi V Rota S Alessio MG Remuzzi G. Accelerating the Depletion of Circulating Anti-Phospholipase A2 Receptor Antibodies in Patients With Severe Membranous Nephropathy: Preliminary Findings With Double Filtration Plasmapheresis and Ofatumumab. Nephron (2020) 144(1):30–5. doi: 10.1159/000501858 31336376

[20] Teisseyre M Boyer-Suavet S Crémoni M Brglez V Esnault V Seitz-Polski B. Analysis and Management of Rituximab Resistance in PLA2R1-Associated Membranous Nephropathy. Kidney Int Rep (2021) 6(4):1183–8. doi: 10.1016/j.ekir.2021.01.022 PMC8071636 33912768

[21] Boyer-Suavet S Andreani M Cremoni M Brglez V Benzaken S Bernard G. Rituximab Bioavailability in Primary Membranous Nephropathy. Nephrol Dial Transplant (2019) 34(8):1423–5. doi: 10.1093/ndt/gfz041 30929012

[22] Jacobs R Langer-Jacobus T Duong M Stahl K Haller H Schmidt RE. Detection and Quantification of Rituximab in the Human Urine. J Immunol Methods (2017) 451:118–21. doi: 10.1016/j.jim.2017.09.001 28890365

[23] Kidney Disease: Improving Global Outcomes (KDIGO) Glomerulonephritis Work Group. KDIGO Clinical Practice Guideline for Glomerulonephritis. (2012), 86–97.

[24] Golay J Semenzato G Rambaldi A Foà R Gaidano G Gamba E. Lessons for the Clinic From Rituximab Pharmacokinetics and Pharmacodynamics. mAbs (2013) 5(6):826–37. doi: 10.4161/mabs.26008 PMC3896596 23933992

[25] Roopenian DC Akilesh S. FcRn: The Neonatal Fc Receptor Comes of Age. Nat Rev Immunol (2007) 7(9):715–25. doi: 10.1038/nri2155 17703228

[26] Kuo TT Aveson VG. Neonatal Fc Receptor and IgG-Based Therapeutics. mAbs (2011) 3(5):422–30. doi: 10.4161/mabs.3.5.16983 PMC3225846 22048693

[27] Lim SH Vaughan AT Ashton-Key M Williams EL Dixon SV Chan HTC. Fc Gamma Receptor IIb on Target B Cells Promotes Rituximab Internalization and Reduces Clinical Efficacy. Blood (2011) 118(9):2530–40. doi: 10.1182/blood-2011-01-330357 21768293

[28] Reddy V Cambridge G Isenberg DA Glennie MJ Cragg MS Leandro M. Internalization of Rituximab and the Efficiency of B Cell Depletion in Rheumatoid Arthritis and Systemic Lupus Erythematosus. Arthritis Rheumatol Hoboken Nj (2015) 67(8):2046–55. doi: 10.1002/art.39167 PMC4737120 25916583

[29] Seitz-Polski B Dahan K Debiec H Rousseau A Andreani M Zaghrini C. High-Dose Rituximab and Early Remission in PLA2R1-Related Membranous Nephropathy. Clin J Am Soc Nephrol CJASN (2019) 14(8):1173–82. doi: 10.2215/CJN.11791018 PMC6682825 31340979

[30] Dahan K Johannet C Esteve E Plaisier E Debiec H Ronco P. Retreatment With Rituximab for Membranous Nephropathy With Persistently Elevated Titers of Anti-Phospholipase A2 Receptor Antibody. Kidney Int (2019) 95(1):233–4. doi: 10.1016/j.kint.2018.08.045 30606419

[31] Waldman M Beck LH Braun M Wilkins K Balow JE Austin HA. Membranous Nephropathy: Pilot Study of a Novel Regimen Combining Cyclosporine and Rituximab. Kidney Int Rep (2016) 1(2):73–84. doi: 10.1016/j.ekir.2016.05.002 27942609 PMC5138549

[32] Van de Logt A-E Dahan K Rousseau A van der Molen R Debiec H Ronco P. Immunological Remission in PLA2R-Antibody-Associated Membranous Nephropathy: Cyclophosphamide *versus* Rituximab. Kidney Int (2018) 93(4):1016–7. doi: 10.1016/j.kint.2017.12.019 29571438

[33] Hofstra JM Beck LH Beck DM Wetzels JF Salant DJ. Anti-Phospholipase A_2_ Receptor Antibodies Correlate With Clinical Status in Idiopathic Membranous Nephropathy. Clin J Am Soc Nephrol CJASN (2011) 6(6):1286–91. doi: 10.2215/CJN.07210810 PMC3109923 21474589

[34] Hofstra JM Debiec H Short CD Pellé T Kleta R Mathieson PW. Antiphospholipase A2 Receptor Antibody Titer and Subclass in Idiopathic Membranous Nephropathy. J Am Soc Nephrol JASN (2012) 23(10):1735–43. doi: 10.1681/ASN.2012030242 PMC3458465 22956816

[35] Jullien P Seitz Polski B Maillard N Thibaudin D Laurent B Ollier E. Anti-Phospholipase A2 Receptor Antibody Levels at Diagnosis Predicts Spontaneous Remission of Idiopathic Membranous Nephropathy. Clin Kidney J (2017) 10(2):209–14. doi: 10.1093/ckj/sfw121 PMC5381233 28396737

[36] Ruggenenti P Debiec H Ruggiero B Chianca A Pellé T Gaspari F. Anti-Phospholipase A2 Receptor Antibody Titer Predicts Post-Rituximab Outcome of Membranous Nephropathy. J Am Soc Nephrol JASN (2015) 26(10):2545–58. doi: 10.1681/ASN.2014070640 PMC4587688 25804280

[37] Seitz-Polski B Dolla G Payré C Girard CA Polidori J Zorzi K. Epitope Spreading of Autoantibody Response to PLA2R Associates With Poor Prognosis in Membranous Nephropathy. J Am Soc Nephrol JASN (2016) 27(5):1517–33. doi: 10.1681/ASN.2014111061 PMC4849812 26567246

